# Corrosion Resistance of Fe-Based Amorphous Films Prepared by the Radio Frequency Magnetron Sputter Method

**DOI:** 10.3390/ma17092071

**Published:** 2024-04-28

**Authors:** Tai-Nan Lin, Pin-Hsun Liao, Cheng-Chin Wang, Hung-Bin Lee, Leu-Wen Tsay

**Affiliations:** 1 Department of Material Research, National Atomic Research Institute, Taoyuan 32546, Taiwan; tnlin@nari.org.tw; 2 Department of Optoelectronics and Materials Technology, National Taiwan Ocean University, Keelung 20224, Taiwan; 11089007@email.ntou.edu.tw (P.-H.L.); lhb6018@mail.ntou.edu.tw (H.-B.L.); 3 Chung Yo Materials Co., Ltd., Kaohsiung 82059, Taiwan; kimwang@cymaterials.com.tw

**Keywords:** R. F. magnetron sputtering, amorphous film, potentiodynamic polarization, XPS

## Abstract

Amorphous thin films can be applied to increase the anti-corrosion ability of critical components. Atomized FeCrNiMoCSiB powders were hot-pressed into a disc target for R. F. magnetron sputtering on a 316L substrate to upgrade its corrosion resistance. The XRD spectrum confirmed that the film deposited by R. F. magnetron sputtering was amorphous. The corrosion resistance of the amorphous film was evaluated in a 1 M HCl solution with potentiodynamic polarization tests, and the results were contrasted with those of a high-velocity oxy-fuel (HVOF) coating and 316L, IN 600, and C 276 alloys. The results indicated that the film hardness and elastic modulus, as measured using a nanoindenter, were 11.1 and 182 GPa, respectively. The principal stresses in two normal directions of the amorphous film were about 60 MPa and in tension. The corrosion resistance of the amorphous film was much greater than that of the other samples, which showed a broad passivation region, even in a 1 M HCl solution. Although the amorphous film showed high corrosion resistance, the original pinholes in the film were weak sites to initiate corrosion pits. After polarization tests, large, deep trenches were seen in the corroded 316L substrate; numerous fine patches in the IN 600 alloy and grain boundary corrosion in the C276 alloy were observed.

## 1. Introduction

A variety of different amorphous alloys (AAs), including Ti- [1,2], Zr- [3,4], Ni- [5,6], and Cu-based [7,8] alloys, have been developed for distinct industries. The absence of microstructural heterogeneity, such as grain boundaries and precipitates, as well as mechanical homogeneity accounts for the remarkable mechanical and chemical properties of AAs [9,10]. Fe-based AAs have many superior characteristics, like excellent corrosion/wear resistances and low costs, among different AAs [11,12,13]. It is reported that FeCrMoCB-Tm AAs have a high glass-forming ability [14,15,16,17,18,19] but high brittleness at room temperature, which greatly restricts their engineering applications. Thus, Fe-based AAs have been provided as powders for thermal spraying [20,21,22,23,24,25] against corrosion and wear for industrial applications [26,27,28].

The chemical compositions, uniform structures, and stable passivation films account for the excellent corrosion resistance of AAs. The addition of Cr has an obviously positive effect on increasing the corrosion resistance of the FeCrNiB AA with a stable passivation film [29]. Forming the more insoluble Cr_2_O_3_ oxide will stabilize and enhance the formation of dense passivation films on FeCrMoCBY coatings [30]. Compared with the FeCrBCP amorphous coating, the addition of a minor amount of Mo (5 at.%) to feedstock powders is advantageous to upgrade the corrosion and wear resistances of coatings [31]. The corrosion resistance of the FeCrMoPCB AA in seawater improves with increasing Cr/Mo content [32]. Furthermore, the addition of C (1–3 wt.%) to FeCuNbSiB amorphous ribbons can significantly improve the corrosion resistance in a 0.1 M H_2_SO_4_ solution [33]. In a 0.5 M KNO_3_ solution, the corrosion potential increases, and the passivation current density decreases with increasing Ni content up to 4 at.% in FeNiCuNbSiB amorphous ribbons [34]. Increasing the crystalline phase in the FeCrMoCBY amorphous coating accounts for a decreased corrosion resistance [35]. The carbide precipitates result in forming a Cr- and Mo-depleted zone and degrade the stability of the passivation film of the FeMoCrYCB amorphous coating [36]. 

Austenitic stainless steels (SSs) are known to be susceptible to chloride stress corrosion cracking (SCC) despite their high general corrosion resistance. Amorphous thin films can be deposited on the critical components against harsh corrosion attack. According to the literature, the characteristics of Fe-based amorphous thin films have rarely been investigated. An Fe-based (FeMoCrYCB) thin film in an artificial sweat solution shows much superior passivation stability relative to 304 SS [37]. In prior work [38], an Fe-based AA (FeCrNiMoCSiB) was used as the feedstock powder for HVOF spraying on a 316L plate. The corrosion rate of the high-velocity oxy-fuel (HVOF) coating is identical to that of the 316L SS in seawater and a 1 M HCl solution, but much higher corrosion-wear resistance was observed in seawater [38]. In this work, an Fe-based AA (FeCrNiMoCSiB) acts as the target material for R. F. magnetron sputtering on 316L SS to upgrade the corrosion resistance of 316L components. The corrosion characteristics of the amorphous film were tested in a 1 M HCl solution with potentiodynamic polarization tests, and the results were contrasted with those of the HVOF coating [38] and 316L [38], IN 600, and C276 alloys. The corroded features of distinct specimens were inspected using a scanning electron microscope (SEM). Moreover, the surface passivation film was analyzed using X-ray photoelectron spectroscopy (XPS).

## 2. Materials and Experiments

In this work, Ar-gas atomized amorphous powders were used for the target material for subsequent sputtering, which Chung Yo Materials Co. offered. The chemical compositions, in weight percentages of the feedstock powders, were 13.65 Ni, 14.41 Mo, 21.53 Cr, 2.30 B, 2.07 C, and 2.73 Si, and the balance was Fe. A 404 F3 high-temperature differential scanning calorimeter (HT-DSC, Netzsch, Selb, Germany) was applied to determine the transformation temperature of the powder. The composition of the C276 alloy was 15.7 Cr, 15.8 Mo, 3.2 W, 6.0 Fe, 0.008 C, 0.6 Mn, and residual Ni. The nominal composition of the IN 600 alloy was the same as that of a Ni alloy with 16 Cr and 8 Fe. The feedstock powders were hot-pressed and sintered into a 6.0 mm plate and then wire-cut to be a 75 mm disc as a target for sputtering. Prior to sputtering, the 316L plate was ground using up to 3000# abrasive paper and then subjected to polishing to a mirror finish. Thin films were deposited on a Si wafer and a mirror-finished 316L plate using an R. F. magnetron sputter system under high vacuum. The sputtering variables included a sputtering power of 120 W, a target-to-substrate distance of 60 mm, a working pressure of 0.7 Pa, and a rotating speed of 5 rpm. Distinct film thicknesses up to 1500 nm could be achieved by altering the deposition time. The film compositions were determined using an electron probe microanalyzer (EPMA, JEOL JXA 8200) equipped with a wavelength-dispersive spectroscope. The phases of the target and the film were detected with a D2 X-ray diffractometer (XRD, Bruker Billerica, MA, USA). The microstructures of the prepared specimens were inspected with a 3400 SEM (Hitachi, Tokyo, Japan). A Hysitron TI 980 nanoindenter (Bruker, Billerica, MA, USA) loaded at 2000 μN was applied to determine the nano-hardness and the Young’s modulus of the prepared film. An optical profiler was used to examine the surface metrology of the film, which provided non-contact surface measurements. A scratch tester (Revetest Scratch Tester: RST^3^) was used to evaluate the thin film’s adhesion property.

The residual stresses of the film deposited on the Si substrate were calculated according to Stoney’s equation [39] as follows:

*σr* = *EH*/6(1 − *ν_S_*)*T_f_R_f_*, (1)

where *σr* is the planar stress in the film; *E, H,* and *ν_S_* are the Young’s modulus (130.2 GPa), the thickness (525 μm), as well as the Poisson’s ratio (0.279) of the Si substrate, respectively; *T_f_* is the film’s thickness; and *R_f_* is the radius of the film’s curvature. 

The potentiodynamic polarization curve of the film was investigated using a standard three-electrode cell system and contrasted with those of the HVOF coating and 316L, IN 600, and C276 alloys. A saturated calomel electrode (SCE) acted as the counter electrode, and the reference electrode was a platinum plate. The potentiodynamic polarization test was carried out in a 1 M HCl solution from −1.0 V to +2.0 V and at a 1 mV/s scanning rate. The elemental depth profile and composition valence of the amorphous film before and after the corrosion test were determined using X-ray photoelectron spectroscopy (XPS, ULVAC-PHI, PHI 5000 Versa Probe) assembled with an Ar^+^ ion etching gun.

## 3. Results and Discussion

**Figure 1:**
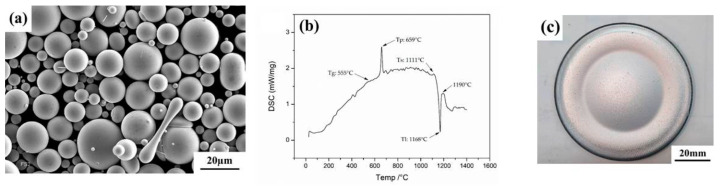
(**a**) The appearance, (**b**) the DSC curve of the feedstock powder, and (**c**) the appearance of the target used for sputtering.

The inherent characteristics of the feedstock are shown in Figure 1, which reveals the appearance and the differential scanning calorimetry (DSC) curve of the powder. The atomized powder used in this work (Figure 1a) predominantly comprised smooth and round particles without a dendritic structure after solidification. As shown in Figure 1b, the DSC curve of the feedstock powder revealed a glass transition temperature (T_g_) of about 555 °C, a peak crystallization temperature (T_p_) of 659 °C, solidus temperature (T_S_) of 1111 °C, and a liquidus temperature (T_L_) of 1168 °C. The solidus temperature of the powder was below 1120 °C. To be used as the target for sputtering, the feedstock powders were pre-vacuumed and hot-pressed at 1080 °C, then the compact powders were sintered for 8 hours into a plate of a 6.0 mm thickness. A 75 mm diameter disc was wire-cut from the sintered plate to be the target for R. F. sputtering (Figure 1c).

**Figure 2:**
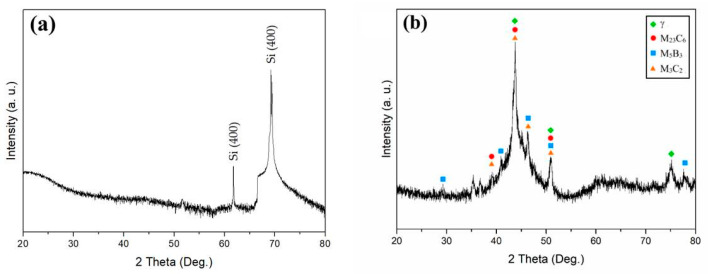
XRD spectra of (**a**) the film (The former is from Si (400) Cu Kβ (1.392 Å), and the latter is from Si (400) Cu Kα (1.54 Å); (**b**) the target.

Figure 2 displays the XRD spectra of the sputtered film and the target. To avoid the interference of the sub-surface layer with the XRD spectrum, the film was deposited directly on the Si wafer. The XRD pattern of the film showed wide and broad peaks in the spectrum (Figure 2a), which indicated the very highly amorphous constituent of the film. By contrast, the XRD pattern of the target (Figure 2b) comprised many sharp peaks, which were identified to be attributed to complicated carbides, borides, and intermetallics formed in the target owing to the slow cooling of the sintered target after hot-pressing the feedstock powders. Although the target was crystalline, the film prepared by R. F. magnetron sputtering was amorphous. Metallic glass films can be fabricated by controlling the sputtering parameters, such as the power and working pressure, so that the as-deposited films inherit the composition and amorphous structure of the target. 

**Figure 3:**
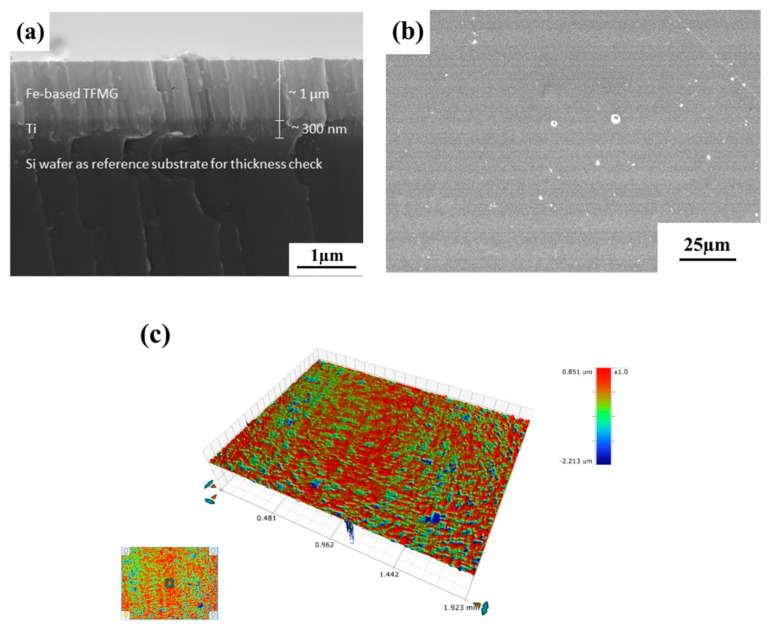
(**a**) The microstructure in the cross-sectional view, (**b**) the surface morphology, and (**c**) the 3D contour profile of the amorphous film.

It was found that peeling was more likely to occur when directly depositing the amorphous film on the 316L substrate, but peeling and/or cracking did not occur on the Si wafer. To avoid the peeling and cracking of the amorphous film, an about 300 nm thick layer of pure Ti was pre-sputtered on the 316L substrate. Figure 3 displays the SEM morphologies of the amorphous film. The amorphous film deposited on the Si wafer, in the cross-sectional view shown in Figure 3a, consisted of a Ti film as the intermediate layer. The chemical compositions of the film, as determined by EPMA, in weight percentages were 16.88 Ni, 13.00 Mo, 21.80 Cr, 1.47 B, 2.38 C, 2.65 Si, and 0.24 Ti, and Fe was the balance. Overall, the deposited film had the about same constituents as the target material. The minor Ti content of the film meant that the yielding volume of the electron beam during EPMA analysis was above the film thickness and reached the intermediate layer. Figure 3b displays the top surface morphology of the amorphous film deposited on the 316L substrate. It was observed that cracking was not found in the film, but some fine particles and pinholes were present in the examined samples (Figure 3b). Those fine particles on the film surface could be from the contaminants present in the chamber. The chemical compositions of those fine particles were inspected using SEM/EDX. The results indicated that most of those fine particles in the examined sample had the about same constituents as the target. It was deduced that those fine particles would be the residues from the target during sputtering. Moreover, those pinholes would be critical defects and would deteriorate the corrosion resistance of the film. The surface texture of the film deposited on the polished 316L substrate was investigated by a 3D contour profiler, as shown in Figure 3c. The Sa, Sp, and Sv values of the film were 20.96 nm, 0.85 μm, and −2.21 μm, respectively. Overall, the amorphous film had a very low surface roughness. 

A nanoindenter was applied to measure the hardness and elastic modulus of the amorphous film, using the sample with the film deposited on the 316 SS. The hardness of the film fell in the range between 10.80 and 11.12 GPa after eight measurements. The elastic modulus of the film was about 181 GPa. The nano-hardness of the film was roughly converted to micro-Vickers hardness values, i.e., from about HV 1102 to 1135. This result revealed that the amorphous film would provide very high wear resistance as compared with traditional engineering alloys. The residual stresses of the amorphous film deposited on the Si substrate were calculated according to Stoney’s equation [39]. Ten measurements were performed at different sites in the film. The principal stresses in two normal directions were calculated according to Stoney’s equation. The two perpendicular residual stresses were in tension and around 60 MPa. The tensile residual stress of the film was low, as compared with the expected strength of the amorphous film [12]. Scratch test experiments determine the practical adhesion strength and mechanical failure modes of hard (HV = 5 GPa or higher) thin (≤30 μm) coatings on metal and ceramic substrates at ambient temperatures according to ASTM C1624-5. In our study, the measured adhesion strength for the as-deposited amorphous film on the stainless-steel substrate was around 25 N at a 5 mm scratch distance, with the scratch load ranging from 50 to 200 N. This result indicates the amorphous film adheres well on the stainless-steel substrate, as expected.

**Figure 4:**
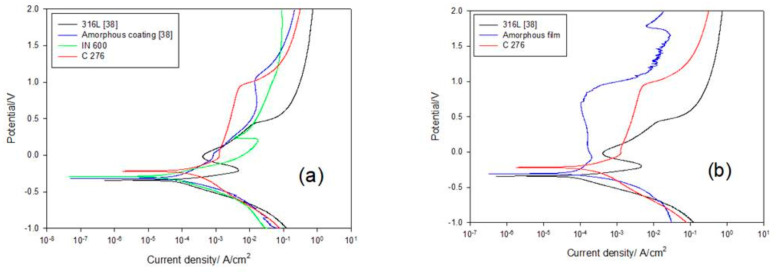
The potentiodynamic polarization curves of the (**a**) IN 600 and C276 alloys in comparison with the 316L and amorphous coating and (**b**) the C276 and amorphous film in comparison with 316L tested in 1 M HCl solution.

Potentiodynamic polarization tests of various specimens were performed at room temperature in a 1 M HCl solution, and the polarization curves are shown in Figure 4. Table 1 lists the corrosion potential (E_Corr_), corrosion current density (i_Corr_), and pitting potential (E_Pit_) of the tested samples. In our prior work [38] studying the corrosion performance of the HVOF coating using the same amorphous powder as used in this work, a 1 M HCl solution was a much more severe corrosion condition relative to seawater and 0.5 M H_2_SO_4_. To distinguish the corrosion resistances among the IN 600, C276, and amorphous film, a 1 M HCl solution is used in this work. Figure 4a reveals the polarization curves of the 316L, IN 600, and C276 alloys and amorphous coating in the 1 M HCl solution. In such a harsh environment, none of the three alloys had an evident passivation region. Among the three alloys, the C276 alloy had a higher E_Corr_ and a lower i_Corr_ than the other two alloys in the 1 M HCl solution. The better corrosion resistance of the C276 alloy was the result of the high alloy contents relative to the other two alloys. The E_Corr_ and i_Corr_ values of 316L were about −0.34 V and 58.2 μA/cm^2^, respectively [38], and around −0.28 V and 29.2 μA/cm^2^ for the IN 600 alloy. This indicated that the IN 600 alloy showed slightly higher corrosion resistance than 316L. Figure 4b displays the potentiodynamic polarization curves of the C276 and 316L alloys and amorphous film in the 1 M HCl solution. This revealed that the amorphous film possessed a marked passivation region and a pitting potential near 1.0 V, whereas the C276 alloy showed an increased current density with increasing potential. Obviously, a passivation zone was not found in the polarization curve of the C276 alloy (Figure 4b). Among the tested samples, only the amorphous film showed a broad passivation zone during corrosion in the 1 M HCl solution. Undoubtedly, the amorphous film provided excellent protection against the harsh HCl attack.

**Figure 5:**
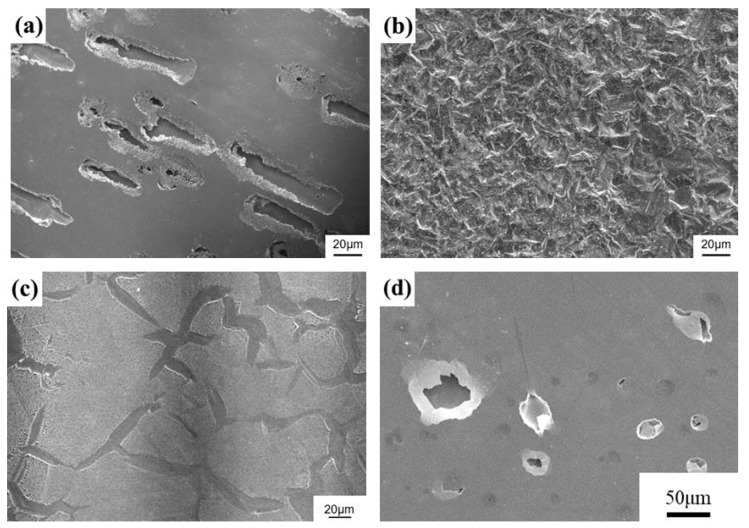
The surface morphologies of the (**a**) 316L, (**b**) IN 600, (**c**) and C276 alloys and (**d**) the amorphous film after polarization tests in the 1 M HCl solution.

The surface morphologies of the distinct test pieces after the polarization tests are displayed in Figure 5. After testing in the 1 M HCl solution, the 316L sample presented severe corrosion owing to the link between fine corrosion pits and corroded trenches (Figure 5a). As compared with the 316L SS, a completely different corroded appearance was seen for the IN 600 alloy tested in the 1 M HCl solution (Figure 5b). The IN 600 alloy showed extensive surface corrosion, which was caused by the extensive dissolution of fine patches in the 1 M HCl solution. By contrast, grain boundary corrosion was more likely to occur for the C276 alloy under the same test conditions (Figure 5c). In addition, the amorphous coating showed fine pits initiated at the interface between residual powders and the molten zone; then, those pits connected to form fine ditches after the polarization test [38]. It was noticed that the corrosion feature of the amorphous film was obviously different from that of the other samples (Figure 5d). Fine pits penetrated the film through pinholes to the Ti buffer layer and grew into a more significant pore, which accounted for the failure of the amorphous film in the 1 M HCl solution (Figure 5d). The original pinholes led to the degraded corrosion resistance of the amorphous film in the harsh HCl environment. 

An Fe-based AA (FeCrNiMoCSiB) was used as the target material and was deposited on 316L SS using R. F. magnetron sputtering. The XRD pattern of the target was composed of peaks attributed to complex carbides, borides, and intermetallics, whereas the deposited film was in the amorphous state. The presence of complex precipitates in the target could be attributed to the slow cooling of the sintered target after hot-pressing. Although the target was crystalline, the film prepared by R. F. magnetron sputtering was amorphous. Metallic glass films can be fabricated by controlling the sputtering parameters, such as the power and working pressure so that the as-deposited films inherit the composition and amorphous structure of the target. In addition, the high cooling rate of the deposited film after sputtering was helpful to form the amorphous film. The elastic modulus of the amorphous film, as determined using a nanoindenter, was about 181 GPa, which agrees with those of Fe-based amorphous alloys [12]. In addition, the amorphous film possessed a nano-hardness from around 10.80 to 11.12 GPa, which meant that the investigated film was expected to have a very high wear resistance. The principal residual stresses were in tension and around 60 MPa. The low principal residual stress could be partly attributed to comparable elastic moduli and thermal conductivities between the 316L and amorphous film. The polarization curve of the amorphous film in the 1 M HCl solution exhibited a marked passivation region and a pitting potential near 1.0 V. In such a harsh solution, only the amorphous film showed a wide passivation region among the tested samples. Although a few pinholes in the film were the cause of the failure in the 1 M HCl solution, the anti-corrosion ability of the amorphous film could be associated with the inherent alloy composition.

**Figure 6:**
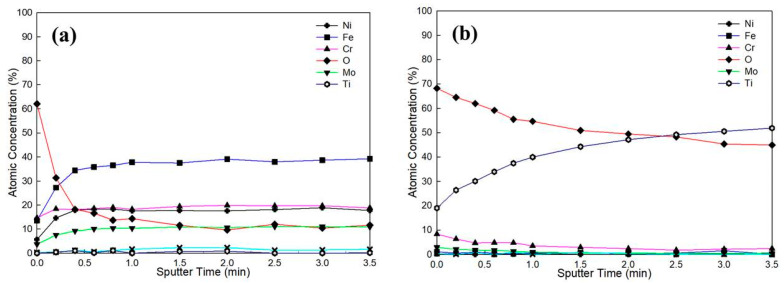
XPS depth profiles of different elements in the amorphous film (**a**) before and (**b**) after corrosion in the 1 M HCl solution.

As displayed in Figure 6 and Table 2, the XPS depth profile (*d*) was achieved using an Ar^+^ ion beam accelerated at 3 keV and a sputtering area of approximately 2 × 2 mm^2^, and the binding energies of the elements, e.g., Fe, Ni, Cr, Mo, Si, Ti, and O, were measured. The etching rate of the film was about 8.16 nm/min for SiO_2_. The charging effect was amended using the C1s peak at 284.6 eV as the baseline for the investigated species [40]. In addition, all the peaks were distinguished based on the NIST database and the results reported in the available literature [41]. As shown in Figure 6a, the O content decreased from 60 at.% to 11 at.% as the depth increased from the surface to the depth after about 1.5 min of sputtering, when the low O content was detected after sputtering. However, after the film was attacked in the 1 M HCl solution, the contents of elements like Fe, Cr, Mo, and Si showed less change, but the Ti content increased with increasing sputtering time. At the depth corresponding to a sputtering time of 3.5 min (Figure 6b), the amorphous film was totally demolished.

**Figure 7:**
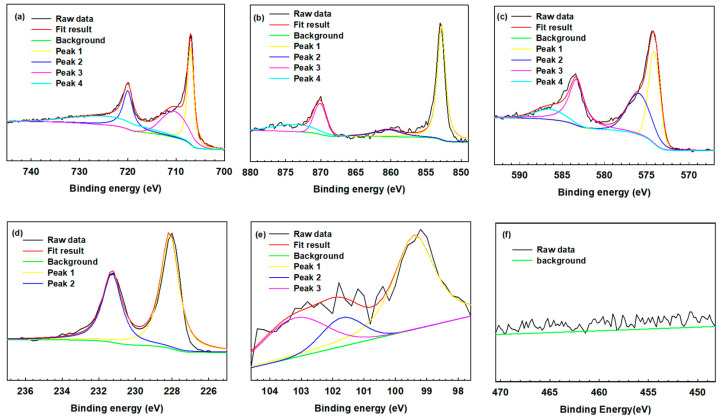
The deconvolution curve-fitting analyses of the binding-energy peaks of the (**a**) Fe, (**b**) Ni, (**c**) Cr, (**d**) Mo, (**e**) Si, and (**f**) Ti elements in the measured XPS spectra of the amorphous film.

**Figure 8:**
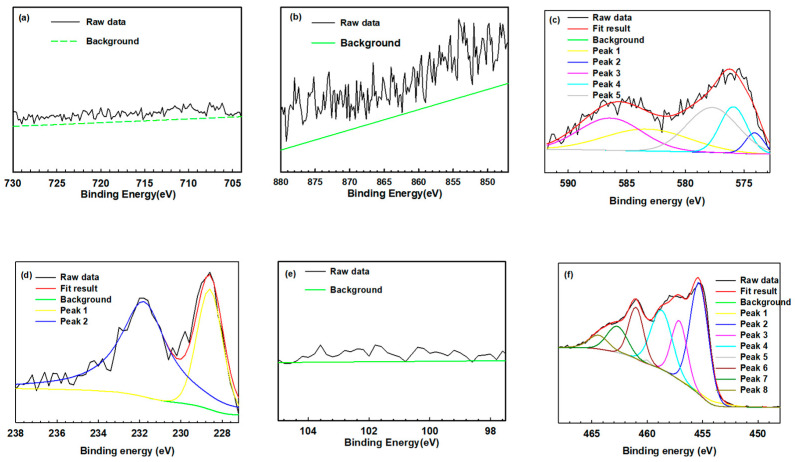
The deconvolution curve-fitting analyses of the binding-energy peaks of the (**a**) Fe, (**b**) Ni, (**c**) Cr, (**d**) Mo, (**e**) Si, and (**f**) Ti elements in the measured XPS spectra of the film after testing in the 1 M HCl solution.

The commercial software XPS Peak version 4.1 was applied to identify the specific peaks, and the binding energy of each species was referenced according to the NIST database, as previous reported. In this sputtered film, elements like Fe, Ni, Cr, Mo, Si, and Ti were analyzed as the primary elements. Table 2 lists the binding energies and spectral peaks corresponding to the relative valences. Figure 7 displays the resolved spectra of the Fe, Ni, Cr, Mo, Si, and Ti elements. Figure 7a reveals the Fe spectra, showing that all the Fe peaks were located at binding energies of approximately 720.0 eV, 707.0 eV, 710.3 eV, and 724.1 eV [42,43,44,45]. Figure 7b shows the Ni spectra, which indicate that all the Ni peaks were located at binding energies of approximately 852.8 eV, 870.1 eV, and 873.6 eV, as well as an Ni^2+^ peak located at 860.4 eV [46,47,48]. As presented in Figure 7c, initially, the Cr peak was located at a binding energy of 583.3 eV, while a Cr peak appeared at 574.1 eV, Cr^3+^ peaks appeared at 575.9 eV and 586.4 eV, and a Cr^6+^ peak appeared at 577.7 eV [42,43,49,50]. Figure 7d displays the initial Mo spectral peaks; they indicate that initially, the Mo in the film was composed of Mo (according to the peak at 228.6 eV) as well as Mo^4+^ (according to the peak at 231.8 eV) [43,49,51,52]. Based on these results, it was deduced that the target used in this work might be contaminated by oxygen during hot-pressing at elevated temperatures and was found to have an O content of 10 at.% (Figure 6a). Figure 7e reveals the Si spectra, showing a Si^0^ peak at 99.4 eV and Si^4+^ peaks at 101.7 eV and 103.2 eV [53,54]. The intensity of the Ti peak was so low that it could be ignored, as shown in Figure 7f. Figure 8 reveals the XPS spectra of the amorphous film after corrosion in the 1 M HCl solution, and the corresponding data are also listed and referenced in Table 2. Because of the failure of the film, the XPS depth profile contained peaks attributed to Ti and residual Cr and Mo. As shown in Figure 8f, the Ti spectra included Ti_2p_ and Ti^0^ peaks at 453.9 eV and 460.1 eV, respectively, as well as Ti^2+^ peaks located at 455.3 eV and 461.0 eV, Ti^3+^ peaks located at 457.1 eV and 462.7 eV, and Ti^4+^ peaks located at 458.7 eV and 464.4 eV [43,55]. Figure 8c shows the Cr core-level spectra and the spectra of the Cr band; most of the Cr remained in the metallic chromium state, with Cr^+3^ peaks at 575.9 eV and 586.4 eV. Chromium might transform to its oxide according to the Cr peak at 574.1 eV and the Cr^+3^ peaks at 575.9 eV and 586.4 eV [42,43], which showed the difference before and after the corrosion test. By contrast, the Cr and Cr^+3^ peaks at 575.9 eV and 586.4 eV, respectively, increased in intensity, which implied the formation of condensed and insoluble Cr_2_O_3_ in the film. Moreover, the Cr_2_O_3_ oxide could effectively prevent Cl- from penetrating the substrate. Figure 8d presents the XPS spectra of the Mo element. According to a study by Hashimoto et al. [56], MoO_2_ can provide substantial corrosion protection because Mo can form a very dense and insoluble oxide relative to the Cr oxide. According to Tian et al. [43], Mo^+6^ can dissolve in water to form MoO_4_^−2^, which can adhere to the surface and prevent Cl- from penetrating the film. In addition, Mo^+6^ effectively avoids the further pitting attack of the corroded iron, which prevents the film from corroding and being demolished. Therefore, the high Cr (above 20%) and Mo (above 14%) contents of the amorphous film ensured the formation of dense and stable Cr and Mo oxides [29,30,31], which were beneficial to form a protective layer, even in the 1 M HCl solution. As revealed in a prior study, the addition of 1–3 wt.% of C to Fe-based AAs could also upgrade their corrosion resistance [33]. In this work, the C content (2.07%) of the investigated film fell within the suggested range. In addition, the 14% Ni content of the film would assist in the formation of Ni oxides, which were also helpful to increase the corrosion resistance of the film. With the addition of 2.73% Si, Ni is reported to increase the Si content in the SiO_2_ passivation film and make the film more protective [13]. As mentioned previously, only a few pinholes were present in the amorphous film, and there was a lack of other defects, like grain boundaries and precipitates, in the film. Therefore, the uniform composition/structure and advantageous alloying made this Fe-based AA film show excellent anti-corrosion ability in a harsh environment.

**Table 2:** Measured and reference binding energies of the XPS spectra generated for amorphous film (a) before and (b) after corrosion.

Before Corrosion			
		Binding Energy (eV)	References
Cr 2p_3/2_	Cr^0^	574.1	[42,51]
	Cr^3+^	575.9	[43]
Cr 2p_1/2_	Cr^0^	583.3	[42]
	Cr^3+^	586.4	
Fe 2p_3/2_	Fe^0^	707.0	[42,43,44]
	Fe^2+^	710.3	[45]
Fe 2p_1/2_	Fe^0^	720.0	[42,43,44]
	Fe^2+^	724.1	[45]
Ni 2p_3/2_	Ni^0^	870.1	[48]
		873.6	[47]
Ni 2p_1/2_	Ni^0^	852.8	[46,47,50]
	Ni^2+^	860.4	
Mo 3d	Mo^0^	228.0	[43,50,53]
	Mo^4+^	231.1	[55]
Si 2p_3/2_	Si^4+^	103.2	[54]
Si 2p_1/2_	Si^0^	99.4	
	Si^4+^	101.7	
After corrosion			
		Binding Energy (eV)	References
Cr 2p_3/2_	Cr^0^	574.1	[50,51]
	Cr^3+^	575.9	[43]
	Cr^6+^	577.7	[51]
Cr 2p_1/2_	Cr^0^	583.3	[42]
	Cr^3+^	586.4	
Mo 3d	Mo^0^	228.6	[43,50,52]
	Mo^4+^	231.8	
Ti 2p_2/3_	Ti^0^	453.9	[43,56]
	Ti^2+^	455.3	
	Ti^3+^	457.1	
	Ti^4+^	458.7	
Ti 2p_1/3_	Ti^0^	460.0	
	Ti^2+^	461.0	
	Ti^3+^	462.7	
	Ti^4+^	464.4	

## 4. Conclusions

(1) With the Ti buffer layer, the amorphous film was successfully deposited on the 316L substrate using R. F. magnetron sputtering, although the FeCrNiMoCSiB target was composed of complex carbides, borides, and intermetallics. The elastic modulus and the nano-hardness of the amorphous film, as measured using a nanoindenter, were about 181 GPa and 11 GPa, respectively;
(2) The polarization curve of the amorphous film in the 1 M HCl solution indicated a broad passivation region and a pitting potential near 1.0 V. The pinholes in the film were the cause of the failure in the 1 M HCl solution after the polarization tests. The XPS spectra of the amorphous film revealed that the dense, insoluble Cr and Mo oxides were beneficial to form a protective layer, even in the 1 M HCl solution. As mentioned previously, only a few pinholes were present in the amorphous film, and there was a lack of other defects, like grain boundaries and precipitates, in the film. Therefore, the uniform composition/structure and advantageous alloying made this Fe-based AA film show excellent anti-corrosion ability in a harsh environment.

## Figures and Tables

**Table 1:** The E_Corr_, i_Corr_, and E_Pit_ values of distinct samples tested in 1 M HCl solution.

Sample	316L [38]	IN 600	Amorphous Coating [38]	C276	Amorphous Film
E_Corr_ (V)	−0.34	−0.28	−0.29	−0.22	−0.3
i_Corr_ (μA/cm^2^)	58.2	29.2	39.2	20.5	58.3
E_Pit_ (V)	0.0	0.25	--	1.04	0.85

## Data Availability

The data are available on request because of restrictions.
